# A Systematic Review of Fear of Cancer Recurrence Among Indigenous and Minority Peoples

**DOI:** 10.3389/fpsyg.2021.621850

**Published:** 2021-05-03

**Authors:** Kate Anderson, Allan ‘Ben' Smith, Abbey Diaz, Joanne Shaw, Phyllis Butow, Louise Sharpe, Afaf Girgis, Sophie Lebel, Haryana Dhillon, Linda Burhansstipanov, Boden Tighe, Gail Garvey

**Affiliations:** ^1^Menzies School of Health Research, Brisbane, QLD, Australia; ^2^Wellbeing and Preventable Chronic Diseases Division, Menzies School of Health Research, Charles Darwin University, Darwin, NT, Australia; ^3^Centre for Oncology Education & Research Translation (CONCERT), Ingham Institute for Applied Medical Research, South Western Sydney Clinical School, University of New South Wales, Sydney, NSW, Australia; ^4^Psycho-Oncology Co-operative Research Group (PoCoG), School of Psychology, Faculty of Science, University of Sydney, Sydney, NSW, Australia; ^5^School of Psychology, University of Ottawa, Ottawa, ON, Canada; ^6^Native American Cancer Research Corporation, Pine, CO, United States

**Keywords:** fear of cancer recurrence, cancer, oncology, review—systematic, minority, Indigenous people

## Abstract

While cancer survivors commonly experience fear and anxiety, a substantial minority experience an enduring and debilitating fear that their cancer will return; a condition commonly referred to as fear of cancer recurrence (FCR). Despite recent advances in this area, little is known about FCR among people from Indigenous or other ethnic and racial minority populations. Given the high prevalence and poor outcomes of cancer among people from these populations, a robust understanding of FCR among people from these groups is critical. The current review identified and aggregated existing literature on FCR amongst adult cancer survivors from Indigenous and minority populations. The protocol of this review was registered with PROSPERO in July 2020 (Registration number: CRD42020161655). A systematic search of bibliographic databases was conducted for relevant articles published from 1997 to November 2019. Data from eligible articles were extracted and appraised for quality by two independent reviewers. Nineteen articles from four countries (United States of America, Canada, Australia and the United Kingdom) met the inclusion criteria, including 14 quantitative, 4 qualitative and 1 mixed-methods study. Only one article reported on an Indigenous population. Few studies reported on FCR prevalence (*n* = 3) or severity (*n* = 9). While the variation in tools used to measure FCR hindered a robust estimate of severity, results suggested some differences in FCR severity between minority and dominant populations, although these may have been due to study metholodological differences. Few factors were reported as being associated with FCR in minorities across multiple studies. The qualitative synthesis found five themes associated with the lived experience of FCR: (i) variations in the lived experience of FCR; (ii) spirituality and worldview impacting on FCR; (iii) the importance of staying positive; (iv) complexities around support; and (v) increasing cancer knowledge. The findings of this review highlight differences in FCR across cultures and contexts, which reinforces the need for culturally-specific approaches to this condition. The dearth of research in this area is of concern given the significant burden of cancer in these populations. A deeper understanding of this condition among Indigenous and minority populations is critical to developing and delivering appropriate and effective psychosocial care for cancer survivors from these groups. Systematic Review Registration: identifier [CRD42020161655].

## Introduction

Increasing clinical and research attention over the past two decades has produced a growing evidence base around *fear of cancer recurrence* (FCR) in people with different cancer types and from diverse populations including some vulnerable groups (Thewes et al., 2012a; Crist and Grunfeld, 2013; Koch et al., 2013; Simard et al., 2013; Almeida et al., 2019). FCR is defined as “*the fear, worry or concern relating to the possibility that cancer will come back or progress”* (Lebel et al., 2016). While some degree of FCR is considered a normal response to the experience of having cancer, estimates suggest that 40–70% of cancer survivors experience a level of FCR that is enduring and debilitating (Thewes et al., 2012a). FCR is associated with varied adverse outcomes in cancer survivors, including: psychological distress; impaired social functioning, and coping with work; reduced quality of life and level of enjoyment; and increased healthcare usage and costs (Avis et al., 2005; Hodges and Humphris, 2009; Lebel et al., 2013; Otto et al., 2018). Moreover, cancer survivors identify FCR as one of their major concerns (Simard et al., 2013).

Several factors are associated with a higher prevalence of FCR, including: younger age; female gender; greater burden of physical symptoms; and psychological factors (Härtl et al., 2003; Simard et al., 2013). Progress has also been made in screening for and assessing FCR (Simard and Savard, 2009), as well as in the development of psychological interventions to reduce FCR, which have been demonstrated to be effective predominantly in patients with early stage cancers treated with curative intent (Sharpe et al., 2017; Hall et al., 2018; Butow et al., 2019; Tauber et al., 2019). Despite these advances, little is known about FCR among cancer survivors from Indigenous, ethnic and racial minority populations and it is unclear whether existing programs and interventions aimed at reducing FCR are appropriate or effective for cancer survivors from these groups. This is surprising, given that cancer is a leading cause of illness and death among many Indigenous populations (United Nations Permanent Forum on Indigenous Issues, 2009; de Souza et al., 2016), and minority populations experience significantly poorer cancer outcomes than other groups with respect to risk factor prevalence, cancer incidence, stage at diagnosis and disease outcomes (United Nations Permanent Forum on Indigenous Issues, 2009; de Souza et al., 2016; Wild et al., 2020), including psychosocial outcomes (Garvey et al., 2020).

Indigenous peoples are recognized as the original inhabitants of a country or geographical region and are known as First Peoples or in some countries by more specific terms such as “*Native American*” in the US, “*First Nations*” in Canada, and “*Aboriginal*” and “*Torres Strait Islander”* in Australia. While there are no globally agreed definitions, the United Nations describes Indigenous peoples as “*inheritors and practitioners of unique cultures and ways of relating to people and the environment. They have retained social, cultural, economic, and political characteristics that are distinct from those of the dominant societies in which they live*” (United Nations, 2020). Similarly, definitions of ethnic and racial minorities are manifold, however, the terms generally refer to ethnic or racial groups in a given country in which they are in a non-dominant position in relation to the dominant population (United Nations, 1992). While there are clear and important distinctions between Indigenous peoples and ethnic and other racial minority populations (hereon respectfully referred to as a collective as IM populations), these groups similarly experience significant disparities in cancer outcomes and share many barriers to accessing and engaging with cancer care (United Nations Permanent Forum on Indigenous Issues, 2009; de Souza et al., 2016).

The current review aimed to explore the following questions:

1. What is the prevalence and severity of FCR among IM populations? Are there differences between IM and dominant populations?
2. What demographic, clinical, social and psychological factors are associated with FCR in IM populations?
3. What are the lived experiences of FCR among IM populations?

The establishment of an evidence base in this specific area will facilitate the evaluation and tailoring of programs and interventions to reduce FCR for cancer survivors from IM populations.

## Methods

### Protocol Registration

A protocol for this systematic review was published on the PROSPERO: International prospective register of systematic reviews website in July 2020 [Registration number: CRD42020161655].

### Search Strategy

The search aimed to identify peer-reviewed literature reporting new empirical data from qualitative, quantitative and mixed-methods studies with a focus on FCR in one or more IM population. The search strategy adhered to the Preferred Reporting Items for Systematic Reviews and Meta-Analyses (PRISMA) guidelines (Moher et al., 2009). The literature search included studies published between 1997 and November 2019. Articles published in scientific journals were identified by searching the bibliographic databases CINAHL, EMBASE, PsycINFO, and PubMed. The search aimed to identify any paper about FCR in adults (18 years or older) from IM populations who have been diagnosed with cancer. There was no restriction on cancer type, cancer stage, time since diagnosis, treatment type, or country/region of residence (see Appendix A for a detailed description of the search strategy).

The search string for Indigenous populations was adapted from a previously published systematic review of Indigenous populations (Angell et al., 2016). The search string for minority populations was adapted from a previously published systematic reviews of minority populations (Dawson et al., 2018).

### Inclusion and Exclusion Criteria

Studies were included if they were: peer-reviewed; published in English; published from 1997 (this start date was chosen as FCR research began to emerge in the literature from this date) to November 2019; report original data from quantitative, qualitative or mixed method studies; involve cancer survivors; and report on the experiences of FCR and/or measures of FCR (e.g., mean FCR score; proportion of participants reporting any/high levels of FCR) separately for at least one IM population, as identified below. All eligible articles were included in the review regardless of quality assessment rating.

Studies were excluded if they were: systematic reviews, meta- and pooled- analyses, reported relative measures only, case studies, case series, commentary, editorial and other opinion papers, even if peer-reviewed; books or book chapters, narrative reviews, conference abstracts, dissertations, and other gray literature; published in languages other than English; or published prior to 1997.

### Data Extraction (Selection and Coding)

All citations identified in the search were downloaded into Rayyan Online Software (Ouzzani et al., 2016). After removal of duplicates, the titles and abstracts were screened separately by two independent reviewers (KA and HH). Reference lists of systematic reviews found in the search were screened to identify other relevant articles for the review. Reviewers resolved disagreements through discussion and evaluation against inclusion/exclusion criteria. Full-text records were retrieved for studies that could not be excluded based on title and abstract alone.

Data extraction was completed by three authors (KA, ABS, and AD), which included: publication information (authors, year of publication, country, region); participant characteristics [IM population(s), cancer type, total number of participants, number of participants per IM group, age, sex]; study characteristics (study design; data collection method, study setting). Study outcomes for the quantitative studies and relevant findings of mixed methods papers were extracted by one author (AD) [FCR measure/s used, statistical tests used, proportion, mean, standard deviation (SD), range, effect size, clinically significant cut offs]. Qualitative and mixed methods papers were imported into NVivo 12 (NVivo qualitative data analysis software; QSR International Pty Ltd. Version 12, 2018) for coding and aggregation by one author (KA).

### Study Quality Assessment

Three authors (KA, ABS, and BT) conducted a critical appraisal of the included articles using the Mixed Methods Appraisal Tool (MMAT) VERSION 2018 (Hong et al., 2018). An overall score out of five was calculated for each article, by tallying how many quality criteria each study was determined to have met.

### Data Synthesis and Aggregation

The quantitative and qualitative syntheses were conducted and presented separately, with the relevant components of any identified mixed methods studies presented in both. Two authors (ABS and AD) synthesized the quantitative data regarding the prevalence, severity, and associated factors of FCR among cancer survivors from IM populations compared to dominant populations (review questions 1 and 2). A meta-synthesis of the qualitative data relating to the *lived experiences* of FCR among cancer survivors from IM populations was conducted by one author (KA).

## Results

**Figure 1:**
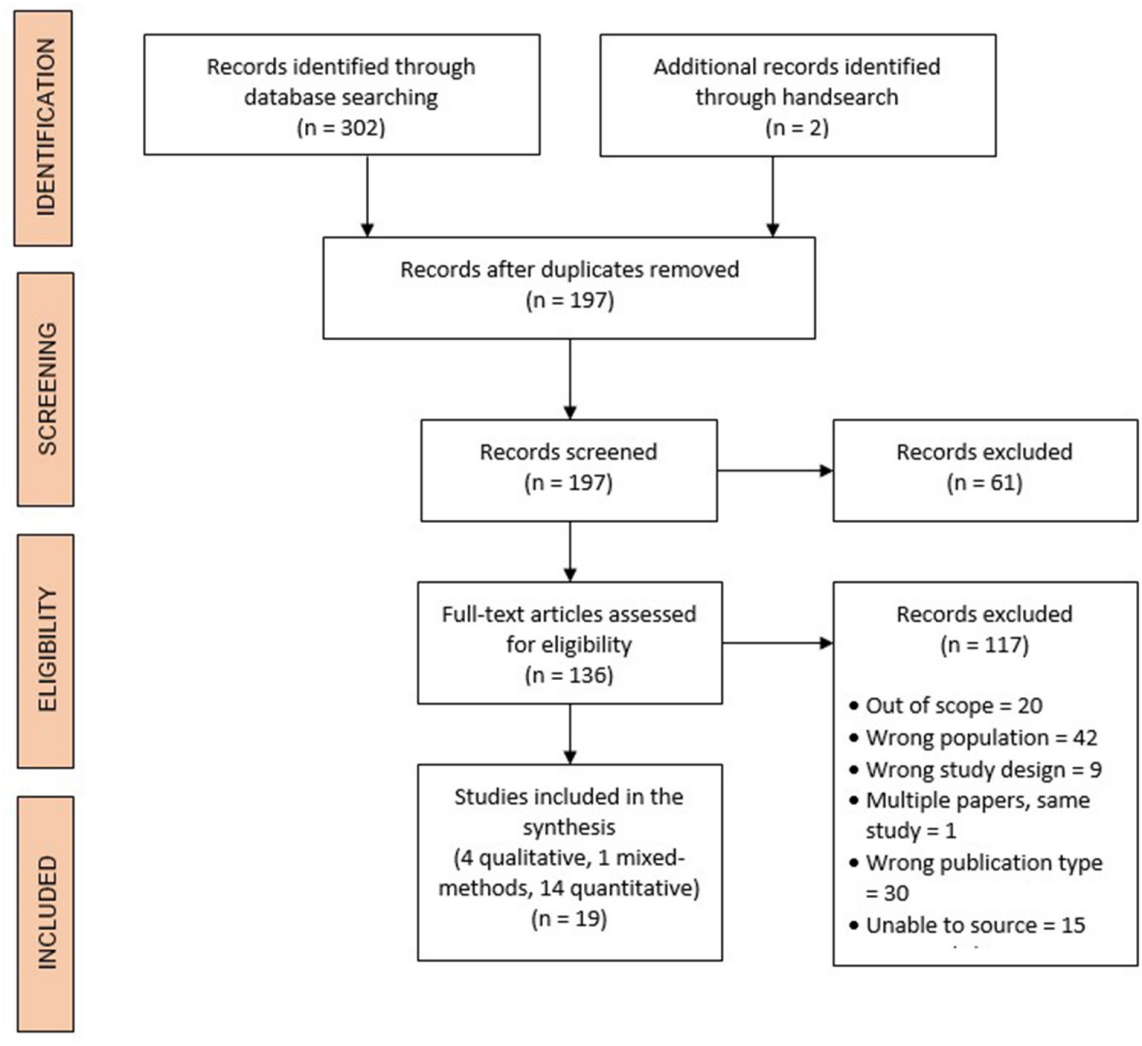
Study selection and PRISMA (Preferred Reporting Items for Systematic Reviews and Meta-Analyses) flow diagram.

Of the 304 records retrieved, 302 were identified in the search and two were identified through a handsearch. From these, 108 duplicates were removed, and 197 records were screened by title and abstract. Of those screened by title and abstract, 136 full-text articles were assessed for eligibility, and 19 records were subsequently considered eligible for inclusion (see Figure 1).

### Characteristics of the Included Articles

A total of 19 studies met inclusion criteria (Braun et al., 2002; Ashing-Giwa et al., 2004; Gill et al., 2004; Krupski et al., 2005; Deimling et al., 2006; Janz et al., 2011, 2016; Liu et al., 2011; Pandya et al., 2011; Bache et al., 2012; Taylor et al., 2012; Butow et al., 2013; Singh-Carlson et al., 2013; Best et al., 2015; Sam, 2016; Ashing et al., 2017; Nápoles et al., 2017; Cho et al., 2018; McMullen et al., 2019). Sixteen studies were conducted in the United States (Braun et al., 2002; Ashing-Giwa et al., 2004; Gill et al., 2004; Krupski et al., 2005; Deimling et al., 2006; Janz et al., 2011, 2016; Liu et al., 2011; Pandya et al., 2011; Taylor et al., 2012; Best et al., 2015; Sam, 2016; Ashing et al., 2017; Nápoles et al., 2017; Cho et al., 2018; McMullen et al., 2019), one in Australia (Butow et al., 2013), Canada (Singh-Carlson et al., 2013) and the United Kingdom (Bache et al., 2012). Fourteen studies were quantitative (Gill et al., 2004; Krupski et al., 2005; Deimling et al., 2006; Janz et al., 2011, 2016; Liu et al., 2011; Pandya et al., 2011; Taylor et al., 2012; Butow et al., 2013; Best et al., 2015; Sam, 2016; Ashing et al., 2017; Cho et al., 2018; McMullen et al., 2019), four were qualitative (Braun et al., 2002; Ashing-Giwa et al., 2004; Bache et al., 2012; Singh-Carlson et al., 2013) and one was mixed-methods (Nápoles et al., 2017). One study included participants from an Indigenous population (Native Hawaiian) (Braun et al., 2002) and all other studies included participants who identified as belonging to one or more minority populations [Latino = 7 (Janz et al., 2011, 2016; Pandya et al., 2011; Sam, 2016; Ashing et al., 2017; Nápoles et al., 2017; McMullen et al., 2019), African American = 11, (Gill et al., 2004; Krupski et al., 2005; Deimling et al., 2006; Janz et al., 2011, 2016; Pandya et al., 2011; Taylor et al., 2012; Best et al., 2015; Sam, 2016; McMullen et al., 2019). Asian American = 4 (Ashing-Giwa et al., 2004; Ashing et al., 2017; Cho et al., 2018; McMullen et al., 2019) and unspecified “non-White” = 1 (Liu et al., 2011) in the United States; first-generation immigrants from various ethnic groups = 1 (Butow et al., 2013) in Australia; South Asian = 1 (Singh-Carlson et al., 2013) in Canada; and African and Black Caribbean = 1 (Bache et al., 2012) in the United Kingdom]. Eleven studies included only breast cancer survivors (Ashing-Giwa et al., 2004; Gill et al., 2004; Janz et al., 2011, 2016; Liu et al., 2011; Taylor et al., 2012; Singh-Carlson et al., 2013; Ashing et al., 2017; Nápoles et al., 2017; Cho et al., 2018); one study each included gynecological (Sam, 2016); colorectal (McMullen et al., 2019); and prostate cancer survivors (Krupski et al., 2005); and five studies included cancer survivors with multiple cancer types (Braun et al., 2002; Deimling et al., 2006; Bache et al., 2012; Butow et al., 2013; Best et al., 2015). The detailed characteristics of the included studies are presented in Table 1 (quantitative studies), Table 2 (qualitative and mixed methods studies). Most quantitative studies used a relevant sampling strategy (12/15) and appropriate statistical analysis (13/15). It was unclear whether the sample was representative of the target population in 10/15 studies, risk of non-response bias was high in 7/15 studies, and only 7/15 studies used a validated FCR measure (see Appendix B for results of the quality appraisal).

**Table 1:** Characteristics of included quantitative studies.

**References**	**Country (region)**	**Study design (data collection)**	**Study setting**	**Participants: n (response rate)**	**IM groups: n (% of total sample)**	**Cancer type: n (%)**	**Time since diagnosis/** **treatment: M (SD)**	**Age in years: M (SD) [range]**	**Sex (%)**
Janz et al. (2011)	USA (Los Angeles, California)	Cross sectional (postal survey)	Cancer registry	2,290 (73%)	Black (*n =* 478; 14.3%) Latina (high acculturation; *n =* 233; 8.1%) Latina (low acculturation; *n =* 256; 8.9%)	Breast (100%)	9 months	56.8 (11.4)	Female (100%)
Cho et al. (2018)	USA (Los Angeles, New York, and Houston)	Cross sectional (postal survey)	Community cultural events, educational conferences and support groups	77 (77.4%)	Chinese American (*n =* 77, 100%)	Breast (100%)	18.8 (11.0) months.	54.4 (8.2) [37–77]	Female (100%)
Gill et al. (2004)	USA (North Carolina)	Cross sectional (telephone survey)	Hospital tumor registries	244 (55% of those contacted, 11.3% attrition)	African American (*n =* 73, 29.9%)	Breast (100%)	Caucasian 6.7 (1.1) years African American 7.0 (1.3) years	64.3 (8.3) [49–87]	Female (100%)
Nápoles et al. (2017)	USA (California)	Cross sectional (telephone survey)	Hospital and community-based cancer support services	118 (51%)	Latinas (*n =* 118, 100%)	Breast (100%)	68% 0–2 years post-diagnosis	54.9 (12.3)	Female (100%)
Liu et al. (2011)	USA (St Louis, Missouri)	Cross-sectional (computer-assisted telephone interview)	Hospital-based cancer treatment service	506 (65.5%)	Non-white (*n =* 98, 19.4%)	Breast (100%)	2 years post definitive breast surgery	58 (10)	Female (100%)
Ashing et al. (2017)	USA (West Coast)	Longitudinal (postal survey at 2 time points)	Community-based health organizations	137 (NR, 34% attrition from T1-T2)	Chinese (51%) Other Asian (49%) - Korean (18.2%) - Filipinas (13.1%) - Vietnamese (6.6%) - Japanese (5.8%) - Mixed (0.7%).	Breast (100%)	T1: mean 2.8 (2.6) years T2: +1 year	54.8 (9.6) [31–83]	Female (100%)
Janz et al. (2016)	USA (Los Angeles, California)	Cross-sectional (postal survey)	Cancer registry	510 survivor-partner pairs (73.1%, 5.4% attrition)	Non-Hispanic Black (*n =* 66; 12.9%) Latina (high acculturation; *n =* 71, 13.9%) Latina (low acculturation; *n =* 62, 12.1%)	Breast (100%)	4 years post-diagnosis	No mean age reported. Under 50 years: *n =* 89, 17.5% 50–65 years: *n =* 263, 51.6% 65 years and over; *n =* 158, 31.0%	Female (100%)
Taylor et al. (2012)	USA (Washington DC)	Cross sectional (postal or in person self-report survey)	University Cancer Centre registry	51 (8.5%)	African American (*n =* 51, 100%)	Breast (100%)	7.2 (4.3) years	64.2 (12.3)	Female (100%)
Krupski et al. (2005)	USA (Los Angeles, California)	Cross-sectional (telephone survey and self-administered questionnaire)	Cancer treatment service	228 (59.2%)	Hispanic (*n =* 115; 50.4%) African American (*n =* 42; 18.4%)	Prostate (100%)	Mean months from biopsy: Caucasian *=* 18.0 Hispanic = 14.6 African American *=* 10.8 Other = 16.8.	Caucasian: 58.98 Hispanic: 63.27 African American: 58.76 Other: 65.12	Male (100%)
McMullen et al. (2019)	USA (6 sites across Colorado, Southern/Northern California, Oregon/Southwest Washington)	Cross sectional (paper, online, and interactive voice response telephone survey)	Patient Outcomes Research to Advance Learning (PORTAL) Network - registries of 6 health systems and 9 research centres affiliated with those systems	1,947 (50.2%)	Hispanic (*n =* 267, 13.7%) Non-Hispanic Asian (*n =* 170, 8.7%) Non-Hispanic Black (*n =* 149, 7.7%) Multiple/other/unknown (*n =* 16, 0.8%)	Colon *n =* 1,418 (72.8%) Rectosigmoid *n =* 101 (5.2%) Rectal *n =* 428 (22.0%)	3.3 (1.1) years	68.6 [17.0–99.2]	Male (51.1%)
Pandya et al. (2011)	USA (Texas)	Cross sectional (face-to-face survey)	National Cancer Institute designated cancer centre	55 (NR)	Hispanic (*n =* 30, 54.5%) African American s (*n =* 5, 9.1%)	Leukemia (100%)	Survival phase: Acute (on treatment): 38.2% Extended (finished treatment/in remission): 40.0% Permanent (cured): 21.8%	54.3 (17.1)	Male (58.2%)
Sam (2016)	USA (Texas)	Cross sectional (web-based survey)	Local clinical facilities, online cancer support groups, Facebook	352 (94%)	Asian 3.7% Hispanic 2.3% African American/Black 1.4%	Ovarian (60%) uterine/endometrial (31%) Cervical (9%)	Disease-free survival M = 5.7 (4.7) years	57.1 (10.1) [29–80]	Female (100%)
Deimling et al. (2006)	USA (Cleveland, Ohio)	Cross sectional (telephone survey)	Cancer registry	321 (48%, 11% attrition)	African American (*n =* 121, 37.7%)	Breast (144, 41.4%) Colorectal (96, 29.9%) Prostate (92, 28.7%).	Med survival 10.4 (5.5) years	72.3 (7.5)	Female (59%)
Butow et al. (2013)	Australia (NSW, QLD, VIC)	Cross sectional (postal survey)	Cancer registries	596 (13.6% of those eligible, 26% of those contacted)	Chinese (*n =* 151, 25.3%) Greek (*n =* 79, 13.3%) Arabic (*n =* 57, 9.6%)	Breast = 204 (34%) Prostate = 154 (26%) Colorectal = 105 (18%) Bladder/kidney = 42 (7%) Leukemia, lymphomas = 45 (8%) Head and neck = 25 (4%) Other = 21 (4%)	Minority 45.8 (24.6) months Anglo 42.9 (20.3) months	Minority: 62.5 (11.0) Anglo: 64.1 (10.7)	Male (51%)
Best et al. (2015)	USA (National)	Cross sectional (secondary analysis of data from survey - modality not specified)	14 state cancer registries	9,006 (35%)	African American (*n =* 933, 10.4%)	Breast (31.5%) Prostate (24.2%) Colorectal (20.8%) Uterine (8.0%) Bladder (6.4%) Melanoma (8.4%) Non-hodgkin lymphoma (0.7%)	2, 5, or 10 years since diagnosis (~one third in each)	67.3 (11.9) [23–100]	Female (55%)

**Table 2:** Characteristics of included qualitative studies.

**References**	**Country (region)**	**Data collection method**	**Study setting**	**IM population/s**	**Cancer type**	**Total participants**	**Participants per ethnic group**	**Age**	**Sex**
Ashing-Giwa et al. (2004)	USA (California)	Focus groups and interviews	Cancer support groups, hospitals, community health clinics	African American, Asian American, Latino American	Breast	102	African Americans (*n =* 24), Asians (*n =* 34), Latinas (*n =* 26) and Caucasians (*n =* 18)	Mean AA = 56 (42–79); Mean Korean *=* 56 (31–78); Mean Chinese = 56 (42–81); Mean Asian = 53 (40–65); Mean Caucasian *=* 55 (43–72); Mean American monolingual = 54 (37–67); Mean Latina = 56 (40–73)	Female
Singh-Carlson et al. (2013)	Canada (BC)	Focus groups and individual interviews	Regional cancer centres	South Asian	Breast	24	South Asian = 24	Mean all partic = 55.5; Range = 28–75	Female
Nápoles et al. (2017)	USA (California)	1) a telephone survey of SSBCS; 2) semi-structured interviews with SSBCS; 3) semi-structured interviews with cancer support providers serving SSBCS	Hospital and Community base cancer support services	Latino American	Breast	143	American Latinos (*n =* 118 in Quant; *n =* 25 in Qual)		Female
Bache et al. (2012)	UK (London)	Semi-structured, in-depth interviews	Charitable support group	African or Black Caribbean	Multiple	8	African (*n =* 1), Black Caribbean (*n =* 5), NR (*n =* 2)	Range = 35–81	Both
Braun et al. (2002)	USA (Hawaii)	PAR; focus groups	Community health centre	Native Hawaiian	Multiple	45	Native Hawaiian (*n =* 45)	Mean all partic = 58 years; Range 36–83 years	Both

### Quantitative Synthesis

#### Prevalence of FCR

Seven studies reported FCR prevalence for IM populations (see Table 3) (Pandya et al., 2011; Taylor et al., 2012; Janz et al., 2016; Ashing et al., 2017; Nápoles et al., 2017; McMullen et al., 2019). The prevalence of FCR ranged widely, from 14% (8/57) of Non-Hispanic Black breast cancer survivors who were 4 years post-diagnosis (Janz et al., 2016) to 67% (34/51) of African American breast cancer survivors at on average 7 years post cancer diagnosis (Taylor et al., 2012) and 67% (20/30) of Hispanic leukemia survivors (Pandya et al., 2011).

**Table 3:** Results from quantitative studies.

**References**	**FCR measure; no. of items [score range]**.	**FCR Prevalence**	FCR Severity^*^ M (SD or 95% CI)	**Factors associated with higher FCR in IM cancer patients and survivors**
Janz et al. (2011)	Study-specific worry about recurrence measure: 3 items [1−5]	NR	White:2.74 Black: 2.47 Latina (high acculturation): 3.08 Latina (low acculturation): 3.78	Sig: compared to whites: higher FCR in latinas lower FCR in blacks
Cho et al. (2018)	Single study-specific item “I worry that my cancer will come back” 0 (not at all) to 4 (very much)	NR	1.75 (1.33)	Sig: worse pain interference, fatigue and emotional well-being Non-sig: pain severity
Gill et al. (2004)	Study-specific checklist of 10 triggers of uncertainty about recurrence and long-term treatment side effects derived from focus groups [0–10]	NR	Average number of triggers per month AA: 1.6 (0.9) White: 1.9 (1.0)	NR
Nápoles et al. (2017)	2 study-specific items “In the past month, how much have you been bothered by: Thoughts that breast cancer will come back Thoughts that will get cancer in other parts of body.” % answering “somewhat/quite a bit/a lot” reported	42%	NR	NR
Liu et al. (2011)	Concern about recurrence scale (CARS) 4 items [1–6]	NR	White: 2.05 (0.99) Non-white: 2.00 (1.35) *t*-test, *p* = 0.71	NR
Ashing et al. (2017)	FACT-B V4 additional item on FCR “I worry about my cancer coming back or spreading” 0 (not at all) to 4 (very much)	T1: 0: “Not at all” = 11.8% 1: “A little bit” = 27.7% 2: “somewhat” = 22.1% 3: “quite a bit” = 17.4% 4: “Very much” = 21.0%T2: 0: “Not at all” = 16.1% 1: “A little bit” = 29.2% 2: “somewhat” = 16.8% 3: “quite a bit” = 13.9% 4: “Very much” = 24.1%	T1: 2.01 (1.36) T2: 1.99 (1.43) *t*-test, *p* = 0.89	Sig: other Asian vs. Chinese ethnicity, Lower health care satisfaction, poorer physical well-being, Poorer emotional well-being, poorer functional well-being, poorer breast cancer-specific HRQOL.Non-sig: age, income, education, language, years living in the US, years since diagnosis, chemotherapy, radiotherapy, cancer stage
Janz et al. (2016)	Study-specific worry about recurrence measure: 3 items [1–5]	Non-hispanic white = 27.1% Non-hispanic black = 14.0% Latina (higher acculturation) = 36.5% Latina (lower acculturation) = 50.0%	NR	Sig: compared to whites: higher FCR in Latinas lower FCR in Blacks
Taylor et al. (2012)	Concern About Recurrence Scale (CARS) 4 items [1–6]	67%	2.65 (1.44)	Sig: Overall FCR related to less time since diagnosis, poorer QOL
Krupski et al. (2005)	Previously used fear of recurrence scale 5 items [0–20]	NR	*High scores = low FCR* Caucasian: 16.31 (4.10) Hispanic: 16.16 (4.80) African American: 18.30 (4.70) Other: 17.06 (4.00)	Sig: compared to caucasians: lower FCR in African AmericansNon-sig: caucasian vs. hispanic
McMullen et al. (2019)	Single study-specific item “What are your biggest health or lifestyle concerns (other than having cancer) since being diagnosed?” 7 identified concerns including 'possibility of cancer recurrence' yes/no response	Concern regarding the possibility of recurrence Non-Hispanic White = 60% Other = 55%	NR	Sig: IM race/ethnicity associated with lower likelihood of FCR
Pandya et al. (2011)	Study-specific item(s) assessing FCR - Specifics not reported	Caucasian = 30% Hispanic = 67% African American *=* 40% Chi-squared, *p* = 0.031	NR	NR
Sam (2016)	Kornblith's FOR Scale 5 items [5–25]	NR	White = 13.79 (4.73; 20.0) Non-White = 11.96 (4.56; 16.0) Mann-Whitney *U*-test, *p* = 0.065	Sig: younger age, negative illness perceptions, greater psychological distressNon-sig: survivorship duration
Deimling et al. (2006)	Cancer Related Health Worry (CRHW) scale 4 items [0–20], higher scores = less worry	NR	11.2 (3.4)	Sig: higher FCR in White/Caucasian vs. Black/African American
Butow et al. (2013)	Cancer Survivors Unmet Needs measure (CASUN) 4 items regarding the future	FCR-related unmet needs: immigrant minorities = 29.6% Anglo-Australians = 17.4%	NR	Sig: need for an interpreter, poorer understanding of the health system, worse anxiety, depression and QOL Non-sig: Immigrant vs. Anglo-Australian background
Best et al. (2015)	Cancer Problems in Living Scale (CPILS) FCR subscale 4 items [0–12]	NR	African American *=* 1.35 (1.45) Non-African American *=* 1.30 (1.39) *t*-test, *p* > 0.05	Sig: Less meaning ^∧^, Less peaceNon-sig: Time since diagnosis†, Cancer stage†

* *Higher score more severe FCR; †Significantly associated with FCR in dominant sample;*

∧ *Not significantly associated with FCR in dominant sample.*

*Sig, statistically significance between-group differences*.

Three studies compared FCR prevalence in minority cancer populations to their Non-Hispanic White/Caucasian counterparts (Pandya et al., 2011; Janz et al., 2016; McMullen et al., 2019), although only two reported FCR prevalence by specific minority group (Pandya et al., 2011; Janz et al., 2016). In these studies, FCR was reported by a greater proportion of Hispanic/Latina people with cancer [Leukemia: (Pandya et al., 2011) 67%; Breast cancer: (Janz et al., 2016) 37% in high acculturated Latina and 50% in low acculturated Latina] than their Non-Hispanic White counterparts [Leukemia 30%; (Pandya et al., 2011). Breast Cancer: (Janz et al., 2016) 27%] (Table 3). Conversely, Janz et al. reported less FCR in Non-Hispanic Black breast cancer survivors compared to Non-Hispanic White breast cancer survivors (14.0 vs. 27.1%) (Janz et al., 2016). While Pandya et al. reported a higher prevalence of FCR in African American than Caucasian Leukemia patients (40 vs. 30%); it is important to note that this study only recruited five African American people (Pandya et al., 2011).

FCR was the most prevalent health/lifestyle concern, emotional concern or symptom among colorectal cancer survivors (McMullen et al., 2019), among Latina breast cancer survivors (Nápoles et al., 2017), and was the highest unmet supportive care need among a mixed sample of immigrant IM cancer survivors (Butow et al., 2013).

#### Severity of FCR

Nine studies reported on the severity of FCR for IM populations (Krupski et al., 2005; Deimling et al., 2006; Janz et al., 2011; Liu et al., 2011; Taylor et al., 2012; Best et al., 2015; Sam, 2016; Ashing et al., 2017; Cho et al., 2018), while one reported on the mean number of triggers of FCR experienced (Gill et al., 2004). FCR was often measured using purpose-designed items, often a single item, making judgements regarding severity difficult, although most mean scores were in the low/moderate range (Table 3). Several studies used the Concerns About Recurrence Scale (CARS) with mean scores ranging from 2.00 (SD = 1.35) in “Non-white” early stage breast cancer survivors (Liu et al., 2011) to 2.65 out of 6.00 (SD = 1.44) in African American breast cancer survivors (Taylor et al., 2012).

Assessment of FCR in distinct IM groups indicate that Hispanic people may experience more severe FCR compared to non-Hispanic Whites. This was observed in older long-term cancer survivors from several cancer groups (Deimling et al., 2006) and survivors of breast cancer (Janz et al., 2011), however no difference was observed between Hispanic and non-Hispanic White non-metastatic prostate cancer survivors (Krupski et al., 2005). In contrast, non-Hispanic Black cancer survivors experienced similar (Best et al., 2015) or less severe (Krupski et al., 2005; Deimling et al., 2006; Janz et al., 2011). FCR compared to non-Hispanic White cancer survivors. One study found Chinese American women reported significantly lower FCR than “other Asian” American women (M = 2.76 years post-diagnosis) (Ashing et al., 2017).

Two studies assessed longitudinal changes in FCR (Janz et al., 2016; Ashing et al., 2017). Higher FCR levels in Hispanic vs. non-Hispanic women 9 months after a breast cancer diagnosis were still evident 4 years post-diagnosis (Janz et al., 2016). Similarly, higher levels of FCR among non-Chinese vs. Chinese Asian-American breast cancer survivors were still evident 1 year post-baseline (Ashing et al., 2017).

#### Factors Associated With FCR

Factors associated with FCR in cancer patients and survivors from minority populations were explored in six studies (Taylor et al., 2012; Butow et al., 2013; Best et al., 2015; Sam, 2016; Ashing et al., 2017; Cho et al., 2018).

##### Demographic and Clinical Correlates

Few consistent relationships emerged between minority peoples' FCR and their demographic or clinical characteristics. Associations with age were assessed in four studies (Taylor et al., 2012; Best et al., 2015; Sam, 2016; Ashing et al., 2017). Age was not associated with FCR in Asian-American breast cancer survivors (Ashing et al., 2017) or overall FCR in African American breast cancer survivors (Taylor et al., 2012), but was negatively correlated with FCR health worries, role worries and death worries domains (Taylor et al., 2012). Age was also negatively correlated with FCR in a heterogenous group of non-White cancer survivors, more so than in Whites (Sam, 2016). Longer time since diagnosis was associated with lower FCR in African American breast cancer survivors (Taylor et al., 2012), but not African Americans with a mix of cancer types (Best et al., 2015) or Non-white gynecological cancer survivors (Sam, 2016). More advanced cancer stage was associated with higher FCR in minority peoples with breast cancer (Ashing et al., 2017), but not in a mixed sample of cancer survivors (Best et al., 2015).

##### Psychological Correlates

Various aspects of minority peoples' perceptions of their illness and its treatment were found to be associated with FCR, although no factors were identified in more than one study. There were a few notable differences in relationships between FCR and psychological variables in White vs. non-White people. Best et al. found a significant negative association between *meaning* and FCR in African American survivors but not in non-African American survivors (Best et al., 2015). Gill et al. found that African American breast cancer survivors were less likely than other survivors to have FCR *triggered by external factors*, namely hearing about somebody else's cancer, environmental triggers or cancer-related media coverage or controversy (Gill et al., 2004). In Australian immigrant minority cancer survivors, more severe unmet FCR-related needs were significantly associated with the need for an interpreter and poorer understanding of the healthcare system (Butow et al., 2013). Among Asian Americans, greater healthcare satisfaction predicted lower subsequent FCR (Ashing et al., 2017). FCR was also found to mediate the impact of pain interference and fatigue on emotional wellbeing (i.e., greater pain interference and fatigue were associated with higher FCR, which was further related to poorer emotional wellbeing) in Chinese American breast cancer survivors (Cho et al., 2018).

### Outcomes of FCR

Several studies found that minority peoples' FCR was associated with worse psychological distress (Butow et al., 2013; Sam, 2016), physical, emotional, functional quality of life and breast cancer specific quality of life (Taylor et al., 2012; Butow et al., 2013; Ashing et al., 2017). Two studies compared outcomes of FCR across minority and dominant populations (Butow et al., 2013; Sam, 2016). FCR and psychological distress demonstrated positive correlations that were moderate in “non-white” and strong in “white” gynecological cancer survivors (Sam, 2016). FCR-related unmet needs and worse anxiety, depression and quality of life were strongly correlated in immigrant Arabic cancer survivors, and moderately correlated in Anglo-Australian and immigrant Chinse and Greek cancer survivors (Butow et al., 2013).

### Qualitative Synthesis

#### Lived Experience of FCR

Of the four qualitative studies (Braun et al., 2002; Ashing-Giwa et al., 2004; Bache et al., 2012; Singh-Carlson et al., 2013) and one mixed-method study (Nápoles et al., 2017) in this review, two were from the USA [California (Ashing-Giwa et al., 2004) and Hawaii] (Braun et al., 2002), one from Canada (British Columbia) (Singh-Carlson et al., 2013) and one from the UK (London) (Bache et al., 2012). The Hawaiian study was the only study in the review that reported on Indigenous participants (Braun et al., 2002). The meta-synthesis undertaken on the qualitative findings reported in these papers revealed five synthesized findings around the lived experience of FCR: (i) variations in the lived experience of FCR; (ii) spirituality and worldview impacting on FCR; (iii) the importance of staying positive and not dwelling on illness; (iv) complexities around family and community support; and (v) increasing cancer knowledge. These synthesized findings are described below, and the meta-synthesis is presented with illustrative quotes in Table 4.

**Table 4:** Summary of main themes identified via meta-aggregation of qualitative studies.

**Themes**	**Key findings**	**Illustrative quotes**
Lived experience of FCR	• FCR is a major concern for some survivors for IM populations • Many negative emotions associated with FCR • FCR is worse than physical effects of cancer • Major concerns around the welfare of their families if they die • Screening tests and symptoms trigger FCR • Some survivors felt abandoned by the health care system after active treatment finishes, which increases anxiety and FCR	“*As a cancer survivor, one of my biggest fear is the 5-year waiting period, to find out if we are going to survive or not. That creates suspense, fear, and negative emotions. Five years is a lot and I never know if I'll be the one winning the battle. I feel like I'm standing on a balance just waiting to see which way it is going to go.” (Latina) (Ashing-Giwa et al., 2004)“ It is not the pain, but it is the anxiety, the fear of it becoming positive. The anxiety is worse than the pain. Pain, I can deal with it. When you're given pain medication, it's relieved. But anxiety, it sticks in your mind.” (Asian American) (Ashing-Giwa et al., 2004)“ As caregivers, we worry about those who depend on us because if something happens to us, who would care and provide for our family, children, parents, etc. who totally depend on us? (Latina) (Ashing-Giwa et al., 2004) Every time I'm due for my mammogram, I can't sleep, worrying. I lose sleep until I get the letter with my results. Then I feel at peace again.” (FocGrp1#9) (Nápoles et al., 2017) “I always worry about mets going to other parts of the body. I do worry, especially when I hear people dying from breast cancer. That hits me; I get really sad.” (Age 41) (Singh-Carlson et al., 2013) “They give surgery, they give you treatments, they say, ‘we got it all.' But you feel a pain, you wonder what is going on, or you feel dizzy. I try not to obsess about it. But I feel it's a legitimate fear. The fact that it might come back and I might have to physically suffer.” (African American) (Ashing-Giwa et al., 2004)“Once they tell you to stop the pills, ‘You're cured, there's nothing wrong with you,' the truth is that one feels, ‘Now what do I do? I have no one to help me.' I felt very abandoned.” (FocGrp1#5) (Nápoles et al., 2017)*
Cultural beliefs and practices impacting on FCR	• Faith and religion were commonly reported as ameliorating FCR • Participants drew strength from involvement in religious, cultural and community groups • Quiet acceptance and prayer were two cultural norms for dealing with difficulty and uncertainty in some IM cultures • Strength among people from IM populations due to shared histories of surviving adversity was seen as an important coping mechanism and ameliorated FCR	“*We all know cancer is a non-curable disease, if we follow up treatment we can get better, but not cured. So who else can decide if we are making it or not, if it's not God? It is only Him who has the last word, and if He decides time has come, no matter what I do, my time is over.” (Latina) (Ashing-Giwa et al., 2004) “I just trust God. Everything trust to God and He will give us miracle. You cannot do anything if you already have cancer. God is the one to give us life, he is the one to get our life.” (Asian American) (Ashing-Giwa et al., 2004) “I draw strength from it because I sing in the choir and you know I pray a lot and feel calm and feel positive from it. It keeps me sane otherwise I don't know.” (South East Asian) (Bache et al., 2012) “I did all the treatment. They did the operation; they did the surgery. What is there to be scared of? You can't do anything about it; just accept it.” (Sikh) (Singh-Carlson et al., 2013) “Every time I have come across a sister with it, I am very proud to say that whatever things that we have went through, we handle it. I think that honestly, it is in our genes. It is in our ancestry. We do this. We get diagnosed with cancer; we have our moments, because I have fallen apart, but we pull ourselves together like a puzzle. I believe that we just have that stamina. We get back up, and dust yourself of and do this kind of thing.” (African American) (Ashing-Giwa et al., 2004)*
Importance of staying positive and not dwelling on illness	• Keeping focused on the positive and not getting caught up in the negative was commonly seen as important to ward of FCR and in surviving cancer more broadly	“*It's too easy to have a negative mind and get depressed and woe is me, I've got cancer, woe woe woe woe woe. I like when people see me, who know my situation, I like when I get the reaction: but you look well though. Whatever life throws at you I believe it's best to just deal with it. You're gonna have your rough days where you're gonna feel like ‘what's the point' but I just feel it's best to just deal with it.” (either African or Black Caribbean – not specified) (Bache et al., 2012)*
Complexities around support from family and community	• Relationships with family were very important for survivors to cope, however, they were sometimes fraught with guilt and misunderstanding • Survivors can feel pressure to hide their FCR to avoid upsetting family members • Survivors sometimes felt that family and community members could not understand their experiences	“*If people who have cancer are in each other's company, they come to know and understand each other, because they get* hounsla *[hope or encouragement] from each other. I will feel better if I talk with similar people who give me* hounsla*.” (South East Asian) (Singh-Carlson et al., 2013) “It is most important to be with people with same disease. Because we understand each other in physical and psychological states. Families don't understand these. They (family members) say they understand, but they expect us to be the same people as before the disease.” (Korean) (Ashing-Giwa et al., 2004)*
Increasing cancer knowledge	• Being informed about cancer and maintaining their treatment regimen were seen by some survivors as beneficial to their recovery from cancer • Some survivors preferred to rely on alternative medicine rather than Western medicine and word of mouth rather than doctors' recommendations	“*Ask for a second opinion. Keep track of everything yourself. The doctor might be more alert knowing that you're keeping track of your own body too. (African American)” (African American) (Ashing-Giwa et al., 2004)*

##### Variations in the Lived Experience of FCR

There were some accounts from cancer survivors from IM populations in these studies about their experiences of FCR, which were contrasted against the experiences of relevant dominant populations and/or other IM groups. Many survivors detailed their experiences of fear, suspense, anxiety and sadness about the thought of their cancer returning (Ashing-Giwa et al., 2004; Singh-Carlson et al., 2013).

Ashing-Giwa et al. found that while cancer survivors from dominant populations in the USA expressed concerns about recurrence associated with incapacitation and loss of autonomy, survivors from African American, Asian American and Latina populations were more concerned about their ability to care for their family if their cancer returned (Ashing-Giwa et al., 2004). Moreover, Ashing-Giwa et al. and Napoles et al. both found that cancer survivors from IM populations in the USA were more likely than dominant populations to experience FCR when obtaining follow up care, including check-ups and mammograms (Ashing-Giwa et al., 2004; Nápoles et al., 2017).

Singh-Carlson et al. found that among female cancer survivors from South Asian populations in Canada, younger women commonly experienced FCR relating to uncertainty around their future, whereas for middle-aged women the FCR centered around what would happen to their children, and older women were not troubled by FCR (Singh-Carlson et al., 2013).

Singh-Carlson et al. reported that IM survivors in Canada were prone to experiencing FCR when hearing stories about other people who are dying from cancer (Singh-Carlson et al., 2013). Ashing-Giwa et al. also found that physical sensations of pain and dizziness were also triggering for FCR among survivors from IM populations in the USA (Ashing-Giwa et al., 2004).

##### Spirituality and Worldview Impacting on FCR

Cancer survivors from IM populations reported spiritual and/or fatalistic beliefs regarding the outcomes of their cancer and their future, which were identified as pivotal in moderating survivors' FCR and fostering psychological adjustment to uncertainties of life after cancer (Braun et al., 2002; Ashing-Giwa et al., 2004; Bache et al., 2012; Singh-Carlson et al., 2013). Across the studies, *God* was commonly described by cancer survivors from IM populations as a source of comfort and as the ultimate decider of one's fate (Bache et al., 2012). Additionally, participation in religious practices, such as attending church services and religious support groups, provided emotional support and distraction, which strengthened cancer survivors' ability to cope with their illness (Bache et al., 2012). Ashing-Giwa et al. found that Asian American survivors relied on their personal sense of faith in managing their fear, African American survivors relied heavily on their prayers and support from their faith community, and Latino-American survivors relied on a combination of faith, prayers and support from their faith community (Ashing-Giwa et al., 2004).

Singh-Carlson et al. found that South Asian cancer survivors in Canada demonstrated a *quiet acceptance* of their cancer experience (Singh-Carlson et al., 2013). This was regarded as reflective of Eastern spiritual beliefs that discourage fighting against suffering and discomfort and instead encourage acceptance and endurance of one's negative experiences (Singh-Carlson et al., 2013). The authors found that among South Asian cancer survivors, belief in *faith* and *karma* were commonly reported to moderate apprehension and reduce fear about their cancer returning (Singh-Carlson et al., 2013).

Similarly, Braun et al. found that Native Hawaiian cancer survivors expressed fatalistic views regarding the outcomes of their cancers, which was thought to foster acceptance and likely reduce FCR (Braun et al., 2002). Ashing-Giwa et al. found that many of the African American survivors drew emotional strength from the long legacy of resilience and survivorship in the history of African Americans (Ashing-Giwa et al., 2004).

##### Staying Positive and Not Dwelling on Illness

Several of the studies in this review found that cancer survivors from IM populations expressed beliefs that maintaining a positive attitude and not dwelling on one's illness are important factors, not just in coping with FCR, but also in overcoming their cancer.

Ashing-Giwa et al. found that a common belief shared across cultural groups was that accepting their illness, but not dwelling on it, was important for coping with cancer (Ashing-Giwa et al., 2004). In order to avoid ruminating on their cancer, older and/or newly emigrated cancer survivors from IM populations, who were often reluctant to seek psychosocial support, distracted themselves from emotional overwhelm with household duties (Ashing-Giwa et al., 2004). Bache et al. also found this to be the case among cancer survivors from IM populations in the United Kingdom (Bache et al., 2012). Survivors attributed emotional and physical resilience to maintaining a positive outlook; and that over-contemplation of illness was thought to accelerate the progression of the cancer (Bache et al., 2012). The researchers postulate that the common avoidance of contemplating cancer, which can lead to missing check-ups and screening and increasing late-detection of cancers, might also be an important component in psychological resilience and a defense against FCR (Bache et al., 2012).

Braun et al. found that many Native Hawaiian cancer survivors had relatives who had died of cancer, which occasioned increased FCR for those people (Braun et al., 2002). This made it difficult to stay positive and some participants took issue with the word *recovery*, as they said: “*You never know. once you get cancer, you might get a recurrence or you might get cancer somewhere else*” (Braun et al., 2002).

Asian and Latina cancer survivors in Ashing-Giwa et al.'s study reported a reliance on inner strength and an emotional response of displacement (e.g., focusing their energy on their families and their household responsibilities) to cope with fear and anxiety around their cancer (Ashing-Giwa et al., 2004). This contrasted with the Caucasian survivors who predominantly drew on a sense of personal empowerment, individual responsibility and knowledge as their source of resiliency (Ashing-Giwa et al., 2004).

##### Complexities Around Family and Community Support

While family and community were identified across the studies as providing critical support for cancer survivors from IM backgrounds to cope with their cancer experiences, there were also commonly identified stressors associated with these relationships.

Two of the included studies reported African American cancer survivors often found strength and emotional support in family and church communities to cope emotionally with their cancer (Ashing-Giwa et al., 2004; Bache et al., 2012). However, Singh-Carlson et al. found that South Asian cancer survivors were ambivalent about receiving emotional support from family and community (Singh-Carlson et al., 2013). The common stigma around cancer in their communities and the prevalent view of cancer as a death-sentence meant that survivors from IM populations were often unwilling to disclose their cancer diagnosis to family and community (Singh-Carlson et al., 2013). This occasioned feelings of isolation and depression among survivors, which heightened rumination and FCR. People who sought support from other cancer survivors had improved *hounsla* (morale) and increased hope for the future (Singh-Carlson et al., 2013).

While support from family was important for many cancer survivors, some studies reported cancer survivors felt great pressure from their families to be positive and well, as they were relied upon to be the traditional caregiver in the family (Braun et al., 2002; Ashing-Giwa et al., 2004; Singh-Carlson et al., 2013). Two studies reported that family and community members did not want to discuss the illness or the survivor's experience with them, and the survivor felt isolated and unsupported (Ashing-Giwa et al., 2004; Singh-Carlson et al., 2013). Braun et al. also found that Native Hawaiian cancer survivors reported stigma and shame around a cancer diagnosis, which caused some people to hide their diagnosis (Braun et al., 2002). These pressures on cancer survivors to not express their negative thoughts and emotions about their cancer to family members sometimes intensified cancer survivors' rumination and FCR.

While the involvement of family and community members occasioned complex and often competing emotional issues, support groups were described by some cancer survivors from IM populations as important to emotionally cope with their illness. Ashing-Giwa et al. reported that these groups offered survivors support via a shared understanding of the experience with other survivors, a lack of pressure to suppress fears and negative feelings, and the stories of survival from cancer providing hope (Ashing-Giwa et al., 2004). These opportunities to share gave survivors some relief from their anxieties and accounts of good cancer outcomes on which they could reflect.

##### Increasing Cancer Knowledge

For some survivors from IM populations, increasing their knowledge about cancer was seen as important in coping and managing FCR—for some people this was via biomedical knowledge and for others it was via traditional medicines and knowledge. Ashing-Giwa et al. found that some cancer survivors relied on alternative medicine and word of mouth rather than rather than Western doctors' recommendations (Ashing-Giwa et al., 2004). Asian American survivors reported that being informed about their illness and maintaining their treatment regimen was beneficial to their recovery from cancer (Ashing-Giwa et al., 2004). Similarly, Bache et al. found that some cancer survivors from IM populations reduced their anxiety by increasing their knowledge about cancer—although it was not known whether such knowledge was philosophically, socially or biomedically based (Bache et al., 2012).

## Discussion

The findings of this review highlight diversity in FCR across different IM populations, which might reflect measurement differences, as well as underlying group differences. While this review found some evidence that FCR might be less prevalence in IM populations that other populations, the lack of culturally-specific FCR measures could account for this apparent difference. The current findings also reveal variability in the factors associated with FCR across IM populations, as well as differences in the lived experience of FCR between different IM populations. Most notably, the findings of our review underscore the paucity of research investigating FCR in IM populations, particularly around the reasons for the varying experiences and outcomes of FCR in minority populations and the near absence of such research for Indigenous populations.

The quantitative synthesis found few consistencies across studies in terms of methods for assessing FCR. The most commonly used validated tool was the *Concerns About Recurrence Scale* (*CARS*) (Vickberg, 2003), whilst others used purpose-designed items. There have been no attempts to develop culturally appropriate measures or to validate existing measures of FCR for any IM populations in these studies. It is notable that the FCRI (Simard and Savard, 2009), which is considered one of the most psychometrically sound FCR measures and has been validated in several different languages (Thewes et al., 2012b), was not used in any of these studies.

The prevalence of FCR in IM populations across the studies ranged from 14 to 67%, which is lower than that reported more broadly (39–97%), but still suggests a substantial proportion of IM cancer survivors suffer from FCR (Simard et al., 2013). Direct comparisons of FCR levels between IM groups and dominant groups were limited, but there was some evidence suggesting higher FCR in Hispanic and lower FCR in African American people compaired with non-Hispanic whites. However, differences between IM and dominant groups were not consistent across studies, which could be due to the different tools used to measure FCR (and lack of validation in IM populations), different ways of grouping ethnicities and races for comparison, different countries and contexts, and different cancer types.

Few consistent relationships were evident between IM populations' FCR and their demographic or clinical characteristics, which is consistent with the FCR literature generally (Smith et al., 2018). There were, however, several psychological factors associated with FCR that were notably different for IM compared with dominant cancer survivors, including meaning and trigger factors. The sense of life meaning and purpose associated with the religious/spiritual beliefs held by many IM survivors may help them engage in more goal-directed action consistent with their values, enabling them to disengage from worries about recurrence (Fardell et al., 2016). Additionally, unmet needs and healthcare satisfaction were associated with FCR differently for some IM populations, as was the mediating effect of FCR on pain interference and fatigue on emotional well-being. It appears that the difficulties navigating the healthcare system and lower levels of healthcare satisfaction experienced more commonly by IM cancer survivors may be exacerbating their FCR, perhaps through the greater sense of general uncertainty that this creates (Fardell et al., 2016; Lebel et al., 2018). The identification of factors associated with FCR in these studies was limited by the fact that the majority of quantitative studies in this review were cross-sectional studies. While some of these were large, they often included only a small IM sub-sample.

The findings of the qualitative meta-synthesis suggest some notable differences in the experience of FCR between cancer survivors across IM groups. This finding is broadly consistent with current thinking that FCR might not be a unique/simple fear but rather a set of different fears, which are often experienced differently between people (Almeida et al., 2019). Despite the many differences, there were some noteworthy parallels in the experience of FCR among cancer survivors across IM populations.

Our qualitative findings suggest that FCR in cancer survivors from IM populations might be commonly moderated via spiritual and/or fatalistic worldviews regarding the outcomes of their cancer and their future. While the particular characteristics of such views differed across cultural groups and individuals, some commonality in the psychological strength and solace that they afforded IM cancer survivors was apparent. While this issue has received little research attention in other populations (Almeida et al., 2019), there is some evidence that a sense of connectedness, which has been characterized as spirituality, helps some breast cancer survivors to adjust and cope post-treatment (Shachar Siman-Tov, 2008). Additionally, our review findings suggest that there is a commonly held belief among IM cancer survivors that maintaining a positive attitude is an important factor in coping with FCR and in overcoming their cancer more generally. This type of thinking is sometimes called the *tyranny of positivity* as it is widely accepted that promoting unsupported beliefs regarding maintaining a positive outlook and avoiding stress will prevent or lessen a person's chances of serious illness are dangerous and likely lead to victim-blaming of those who are ill for not being positive enough (Aspinwall and Tedeschi, 2010). While staying positive may assist coping and reduce FCR for some IM cancer survivors, the promotion of positivity to IM survivors should be balanced with consideration of the potential negative effects of overemphasizing its import.

Our qualitative findings around the complexities associated with family and community support for cancer survivors from IM populations were notable. Strong family and community networks are commonly identified as important supports for cancer patients in many IM populations, the pressure and stigma that these networks can occasion for cancer survivors might sometimes serve to heighten FCR, as it prevents cancer survivors from expressing their fears in order to protect those around them (Soriano et al., 2018). Sharing concerns with social supports may help normalize concerns, while internalization of fears may lead to greater rumination and worsening of FCR.

Taken together, the results of our review revealed that FCR is experienced differently across IM populations, which is perhaps unsurprising, considering the diversity of cultural groups, geographic and social contexts and study methods, as well as the documented variability in FCR levels across other populations generally (Almeida et al., 2019). Spirituality, family and community support, and need for cancer information have such marked but variable roles in the experience of FCR for cancer survivors from different IM populations that approaches to reducing FCR must be flexible and adaptable enough to meet each survivor's individual circumstances and needs.

Our review highlights important considerations for future FCR research and practice addressing key priorities [e.g., better FCR detection/screening and more accessible FCR treatment models (Shaw et al., 2021)], to ensure that IM experiences and needs regarding FCR are accommodated. To enable FCR screening in IM populations, further work is needed to validate brief FCR measures in IM populations and adapt them where needed. To date, no interventions specifically targeting FCR in an IM population have been trialed (Tauber et al., 2019). To make FCR interventions accessible and engaging for IM populations it is essential that their diverse cultural beliefs and norms be considered. For instance, interventions incorporating elements of acceptance and commitment therapy may appeal more to IM populations where spiritual beliefs around acceptance are common. Given the importance of community support for many IM populations, but occasionally negative impact of community expectations, delivery of interventions focused on normalizing and coping with FCR, not just for survivors, but also their caregivers and communities, may be beneficial. Our findings make clear that effective measurement and treatment of FCR must take into account the individual and cultural circumstances of cancer survivors. While patient-reported outcome measures are commonly translated for culturally and linguistically diverse respondents, this approach fails to capture critical issues relevant to specific populations. Measures of FCR that include items developed by and with people from IM populations will offer the most effective means of identifying IM patients' concerns associated with this condition. Considering the variable experience of FCR across different IM populations, it is essential that researchers and clinicians partner with representatives of the specific IM communities they are serving, to ensure research and clinical practice is culturally responsive.

### Limitations and Future Directions

There are methodological limitations of our review that must be noted. The heterogeneity in the study designs, samples, cancers and methods for assessing FCR across the studies included in this review only enable the aggregation of descriptive statistics. Further, limited evidence was available regarding factors associated with FCR and how these differed between IM and majority populations. This limits the strength of the evidence that can be presented and elicits more questions than answers. As this review aims to establish an evidence base within an under-researched area, this limitation is to be expected.

Most studies included in this review were conducted in the US, which highlights the need for greater research attention to this issue in other countries. The single study reporting on FCR for Indigenous people (also from the US), while offering a valuable insight into the condition for this particular group, cannot reflect the experience of survivors across different Indigenous populations. Given this limitation, it is tenuous to make any generalizations about FCR for other Indigenous populations.

This review is a first attempt to draw attention to the dearth of literature around FCR for cancer survivors from IM populations. The findings of this review are intended to identify the areas in greatest need of research attention. Most notable is the lack of research into FCR among Indigenous cancer survivors. The fact that all papers identified in our review were in Anglophile countries highlights the paucity of FCR research internationally. Fortunately, the number of articles published on FCR in IM populations appears to be increasing, with an updated search in March 2021 finding seven further relavent papers. We hope this review will stimulate further research in the area and that an update of this review would incorporate many more papers. The development of culturally-appropriate measures of FCR, or the validation of existing measures of FCR for IM populations, would also aid further research. Ensuring that research in this space is conducted by and/or with researchers from IM populations is imperative.

### Conclusions

This review highlights the potential impact of culture and context on FCR and reinforces the need for a culturally-specific lens to be used in consideration and measurement of this condition. The paucity of research investigating FCR among cancer survivors from Indigenous groups requires urgent attention.

## Data Availability Statement

This paper presents findings from our synthesis of previously reported findings. Each study included in our review is included in our reference list. For original data enquiries, please contact the corresponding authors of these papers.

## Author Contributions

KA, ABS, and AD participated in research design, writing of the paper, performance of the research, and data analysis. JS, LS, AG, SL, HD, LB, and PB participated in research design and writing of the paper. BT participated in writing of the paper and data analysis. GG participated in research design, writing of the paper, performance of the research, and data analysis. All authors contributed to the article and approved the submitted version.

## Conflict of Interest

The authors declare that the research was conducted in the absence of any commercial or financial relationships that could be construed as a potential conflict of interest.
