# Supplementary material for: A Systematic Review of Fear of Cancer Recurrence Among Indigenous and Minority Peoples
Source: Front Psychol. 2021 May 3;12:621850. doi: 10.3389/fpsyg.2021.621850 (PMC8126623; doi:10.3389/fpsyg.2021.621850)
Supplement: Supplementary file 1 [file Data_Sheet_1.docx]

**Appendix A – Detailed search strategy**

**Population:** People from IM populations who have been diagnosed with cancer and are 18 years or older.

**Main outcome(s):** Fear, worry, or concern about cancer returning or progressing (collectively FCR). Outcomes that include, but not limited to, FCR, such as uncertainty or worry about the future, will also be included.

1. The population terms were informed reviews conducted by Dawson et al. (2018) and by Angell et al. (2016). The FCR-related search terms were informed by a review conducted by Thewes et al. (2012) and a protocol for a review authored by Leske et al. (2018). Thewes and colleagues aimed to identify all available FCR assessment instruments and critically appraise them. This review found that FCR has not been routinely assessed in studies about cancer survivorship. Leske and colleagues’ protocol for a systematic review seeks to understand the prevalence and impact of FCR in families and caregivers of cancer patients. Appropriate Subject Headings and MeSH terms were used in our search.

Following is a detailed list of search terms:

- **Population terms:** 'American Indian*' OR Eskimo* OR 'First Nation*' OR 'First people*' OR Indigenous OR Inuit* OR Iñupiat* OR Inuvialuit* OR Maori* OR 'Native American*' OR 'native people*' OR 'native population' OR 'native Canadian' OR Navajo OR Navaho OR Nunangat* OR tribe OR tribal OR Yuit OR Yupik OR Zuni OR 'Indigenous population*' OR Aboriginal OR 'Torres Strait Islander' OR Metis* OR ethnic* OR race OR cultur* OR minorit* OR immigrant OR migrant OR CALD OR underserved OR vulnerable OR ‘Sami’ OR ‘Sampi’ OR ‘Lapland’ OR ‘Pacific Islander’ OR ‘Pasifika’
- **FCR terms:** fear* OR concern* OR anxiet* OR worr* OR recur* OR relapse OR coming back OR progress*OR reoccur* OR spread
- **Disease terms:** cancer* OR neoplasm* OR tumour* OR tumor*

***References***

Angell B, Muhunthan J, et al. The health-related quality of life of Indigenous populations: a global systematic review. Qual Life Res 2016;25(9):2161-78.

Dawson S, Campbell SM, et al. Black and minority ethnic group involvement in health and social care research: A systematic review. Health Expect 2018;21(1):3-22.

Leske S, Smith AB, et al. A protocol for an updated and expanded systematic mixed studies review of fear of cancer recurrence in families and caregivers of adults diagnosed with cancer. Systematic Reviews 2018;7(1):134.

Thewes B, Butow P, et al. Fear of cancer recurrence in young women with a history of early-stage breast cancer: a cross-sectional study of prevalence and association with health behaviours. Support Care Cancer 2012;20(11):2651-9.

***Appendix B – Mixed Methods Appraisal Tool Study Quality Appraisal Scores***

| **QUANTITATIVE STUDIES** | | | | | | |
| --- | --- | --- | --- | --- | --- | --- |
| **Authors**  **(Year)** | **Is the sampling strategy relevant to address the research question?** | | **Is the sample representative of the target population?** | **Are the measurements appropriate?** | **Is the risk of nonresponse bias low?** | **Is the statistical analysis appropriate to answer the research question?** |
| Janz at al (2011)^37^ | Y | | ? | ? | Y | Y |
| Cho et al (2018)^34^ | Y | | Y | ? | Y | Y |
| Gil et al (2004)^36^ | Y | | ? | ? | N | Y |
| Napoles et al (2017)^42^ | Y | | ? | ? | N | Y |
| Liu et al (2011)^40^ | Y | | Y | Y | Y | Y |
| Ashing et al (2017)^29^ | Y | | ? | Y | Y | Y |
| Janz et al (2016)^38^ | Y | | ? | ? | Y | Y |
| Taylor et al (2012)^45^ | Y | | Y | Y | N | Y |
| Krupski et al (2005)^39^ | Y | | ? | Y | Y | Y |
| McMullen et al (2019)^41^ | Y | | ? | ? | ? | Y |
| Pandya et al (2011)^43^ | ? | | ? | ? | ? | ? |
| Annamma (2016)^28^ | N | | ? | Y | ? | Y |
| Deimling et al (2006)^35^ | Y | | Y | ? | N | Y |
| Butow et al (2013)^46^ | Y | | ? | Y | Y | Y |
| Best et al (2015)^32^ | Y | | Y | Y | Y | Y |
| **QUALITATIVE STUDIES** | | | | | | |
| **Authors**  **(Year)** | **Is the qualitative approach appropriate to answer the research question?** | **Are the qualitative data collection methods adequate to address the research question?** | | **Are the findings adequately derived from the data?** | **Is the interpretation of results sufficiently substantiated by data?** | **Is there coherence between qualitative data sources, collection, analysis and interpretation?** |
| Ashing-Giwa et al (2004)^30^ | Y | Y | | Y | Y | Y |
| Singh-Carlson et al (2013)^44^ | Y | Y | | Y | Y | Y |
| Napoles et al (2017)^42^ | Y | Y | | Y | Y | Y |
| Bache et al (2012)^31^ | Y | Y | | Y | Y | Y |
| Braun et al (2002)^33^ | Y | Y | | Y | Y | Y |
| **MIXED-METHOD STUDIES** | | | | | | |
| **Authors**  **(Year)** | **Is there an adequate rationale for using a mixed methods design to address the research question?** | **Are the different components of the study effectively integrated to answer the research question?** | | **Are the outputs of the integration of qualitative and quantitative components adequately interpreted?** | **Are divergences and inconsistencies between quantitative and qualitative results adequately addressed?** | **Do the different components of the study adhere to the quality criteria of each tradition of the methods involved?** |
| Napoles et al (2017)^42^ | **?** | **Y** | | **Y** | **?** | **Y** |

Abbreviations: Y = Yes, N = No, ? = Cannot tell
